# Hornet Sting-Induced Type I Kounis Syndrome Presenting as a Non-ST-Elevation Acute Coronary Syndrome: A Case Report

**DOI:** 10.7759/cureus.115847

**Published:** 2026-09-06

**Authors:** Rajae Zidouh, Souad Abbi, Suzan Iskandar, Nada Tababi, Taha Hassani

**Affiliations:** 1 Department of Cardiology, Ibn Sina University Hospital Center, Mohammed V University, Rabat, MAR; 2 Department of Cardiology, Bahman Hospital, Beirut, LBN; 3 Department of Cardiology, Centre Hospitalier de Rodez, Hôpital Jacques Puel, Rodez, FRA

**Keywords:** allergic acute coronary syndrome, anaphylaxis, coronary vasospasm, epinephrine, hornet sting, kounis syndrome, myocardial ischemia

## Abstract

Kounis syndrome is an acute coronary syndrome triggered by an allergic or anaphylactic reaction. Diagnosis can be challenging when epinephrine is administered during the same episode because both the allergic reaction and epinephrine may influence coronary vascular tone.

We report the case of a 62-year-old man with no previous cardiac history who developed anaphylactic shock after a hornet sting to the upper lip. Acute constrictive chest pain began during the allergic reaction, before epinephrine administration. He was promptly treated with 0.55 mg of epinephrine, methylprednisolone, and dexchlorpheniramine. An electrocardiogram obtained during the acute episode showed ST-segment depression in leads III, aVF, and V3-V6. The chest pain and electrocardiographic abnormalities subsequently resolved. Troponin peaked at 208 ng/L. Transthoracic echocardiography showed preserved left ventricular systolic function without regional wall-motion abnormalities. Coronary angiography revealed only minor non-obstructive coronary irregularities, with no culprit lesion, and follow-up cardiac magnetic resonance imaging was normal. The overall presentation was consistent with type I Kounis syndrome, most likely caused by transient allergy-mediated coronary vasospasm. The onset of chest pain before epinephrine administration was an important element supporting the allergic coronary event as the primary mechanism.

## Introduction

Kounis syndrome describes acute myocardial ischemia occurring in the setting of an allergic or anaphylactic reaction. The concept was originally described as allergic angina caused by histamine-mediated coronary spasm [1]. It is now understood as a clinical spectrum in which mast-cell activation and the release of inflammatory mediators can produce coronary vasospasm, destabilize an atherosclerotic plaque, or contribute to coronary thrombosis [2,3].

Three main variants are traditionally recognized. Type I is characterized by allergy-mediated coronary vasospasm in patients with normal or near-normal coronary arteries and may occur with or without an increase in cardiac biomarkers. Type II occurs in patients with pre-existing coronary atherosclerosis, in whom the allergic response may trigger vasospasm or plaque disruption. Type III is associated with hypersensitivity-related stent thrombosis [2,3].

Hymenoptera stings are well-established triggers of Kounis syndrome [4]. The diagnosis can become particularly challenging when epinephrine is required for anaphylaxis because the treatment itself has cardiovascular effects. We report a type I Kounis syndrome after a hornet sting, presenting as a non-ST-elevation acute coronary syndrome. The timing of the chest pain in relation to the allergic reaction and epinephrine administration was central to the final interpretation.

## Case presentation

A 62-year-old man with no previous cardiovascular disease was transferred to our Cardiology Department after an anaphylactic reaction caused by a hornet sting to the upper lip. His medical history was notable for obstructive sleep apnea treated with positive airway pressure. He was taking no regular medication. He had a previous hypersensitivity reaction to amoxicillin and amoxicillin-clavulanate, described as early angioedema, as well as a history of urticaria after certain foods and seasonal pollen allergy.

Immediately after the sting, the allergic reaction progressed to anaphylactic shock and was accompanied by acute constrictive chest pain. The chest pain began before epinephrine administration. As part of the emergency management of anaphylaxis, the patient promptly received 0.55 mg of epinephrine together with methylprednisolone and dexchlorpheniramine. An electrocardiogram obtained during the acute episode showed ST-segment depression in leads III, aVF, and V3-V6 (Figure 1). The exact timing of the ECG relative to epinephrine administration could not be established from the available records. Following treatment, the allergic manifestations improved, the chest pain resolved, and the ST-segment abnormalities regressed.

**Figure 1:**
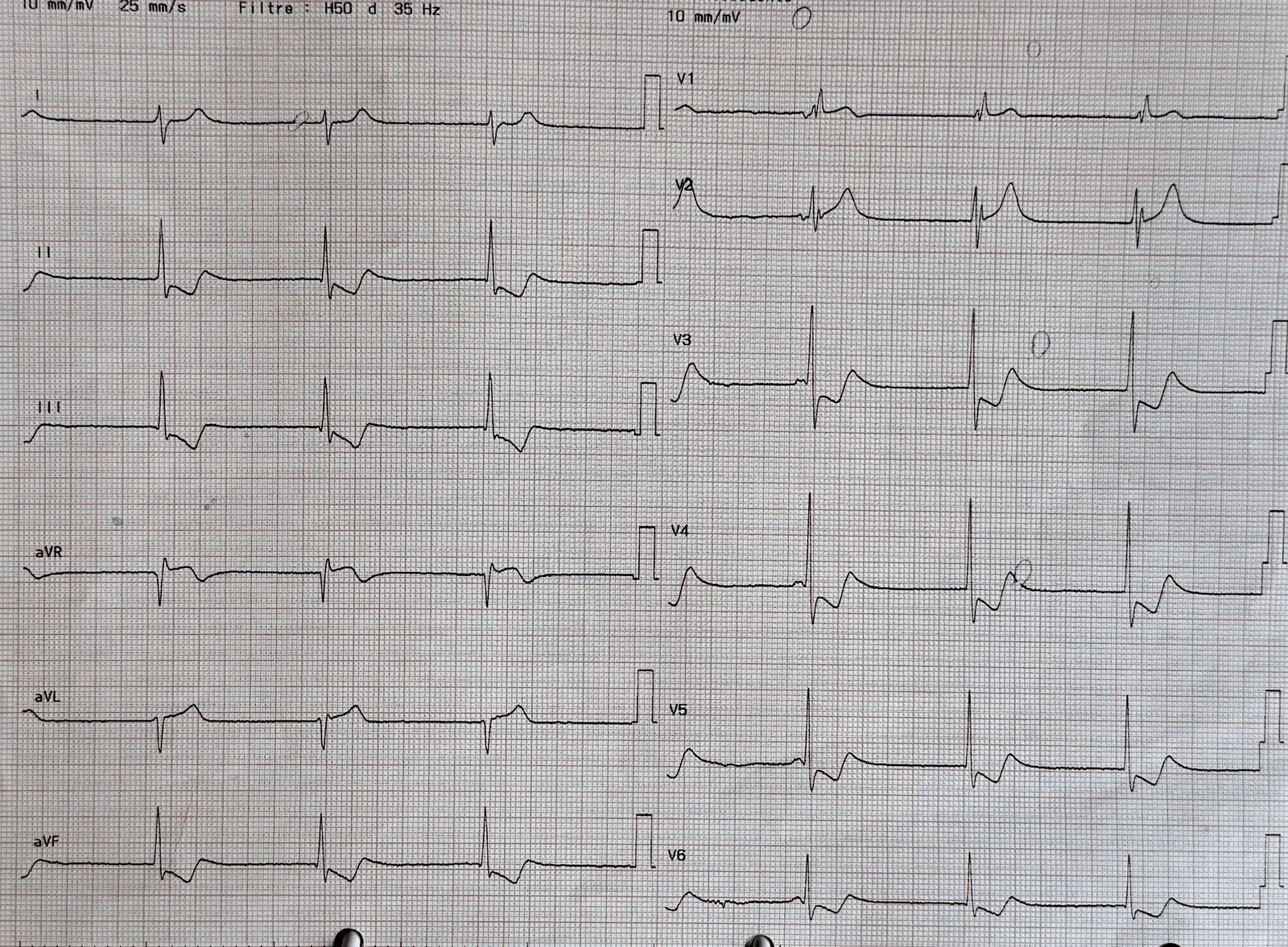
Initial electrocardiogram obtained during chest pain following the hornet sting showing ST-segment depression in leads III, aVF, and V3-V6

At transfer, the patient was afebrile and hemodynamically stable, with a blood pressure of 122/69 mmHg and oxygen saturation of 98% on room air. Intermittent bradycardia was observed, with a lowest recorded heart rate of 40 beats/min. Cardiovascular examination showed regular heart sounds without an audible murmur, and pulmonary auscultation was clear. A subsequent 12-lead ECG showed sinus rhythm at 73 beats/min, a PR interval of 136 ms, QRS duration of 100 ms, and a corrected QT interval of 398 ms, without persistent ischemic repolarization abnormalities. An incomplete right bundle branch block pattern was present (Figure 2).

**Figure 2:**
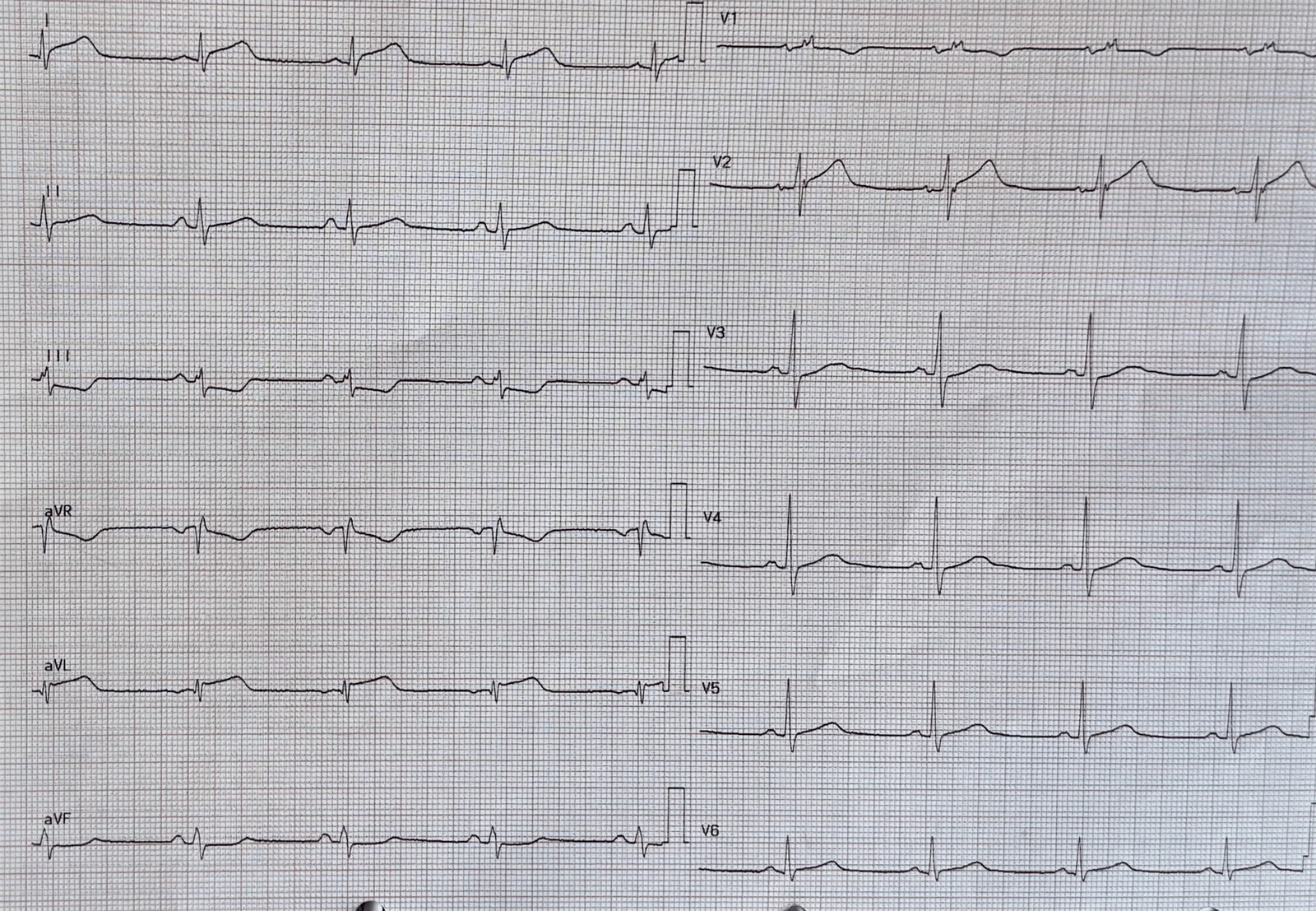
Follow-up electrocardiogram showing sinus rhythm and regression of the ischemic ST-segment abnormalities

Laboratory investigations are summarized in Table 1. Hemoglobin was 15.2 g/dL, the platelet count was 157 × 10⁹/L, and creatinine was 77 µmol/L, with no clinically significant hepatic or electrolyte abnormality. C-reactive protein was 10 mg/L. Troponin reached a peak of 208 ng/L (reference range: 0-14 ng/L) and subsequently decreased. NT-proBNP was 186 pg/mL. D-dimer was >4000 µg/L (reference range: <500 µg/L), prompting contrast-enhanced chest computed tomography prior to coronary angiography, which excluded pulmonary embolism.

**Table 1:** Laboratory investigations

Parameters	Patient values	Reference range
Hemoglobin	15.2 g/dL	13.0-17.0 g/dL
Platelet count	157 × 10⁹/L	150-400 × 10⁹/L
Creatinine	77 µmol/L	62-106 µmol/L
C-reactive protein	10 mg/L	<5 mg/L
Troponin, peak	208 ng/L	0-14 ng/L
NT-proBNP	186 pg/mL	<900 pg/mL for acute heart failure; <125 pg/mL for chronic heart failure
D-dimer	>4000 µg/L	<500 µg/L
Troponin, follow-up	67 ng/L	0-14 ng/L

Transthoracic echocardiography showed a non-dilated, non-hypertrophied left ventricle with preserved global and regional systolic function and no wall-motion abnormality. Right ventricular systolic function was preserved. There was no significant mitral or aortic valve disease and no pericardial effusion.

Coronary angiography was performed the following day. The coronary circulation was right dominant. The left main coronary artery had only a minor irregularity of less than 30%. The mid left anterior descending artery and proximal circumflex artery also showed non-significant irregularities of less than 30%, while the dominant right coronary artery was free of significant stenosis. No culprit lesion or obstructive coronary disease requiring revascularization was identified (Figure 3).

**Figure 3:**
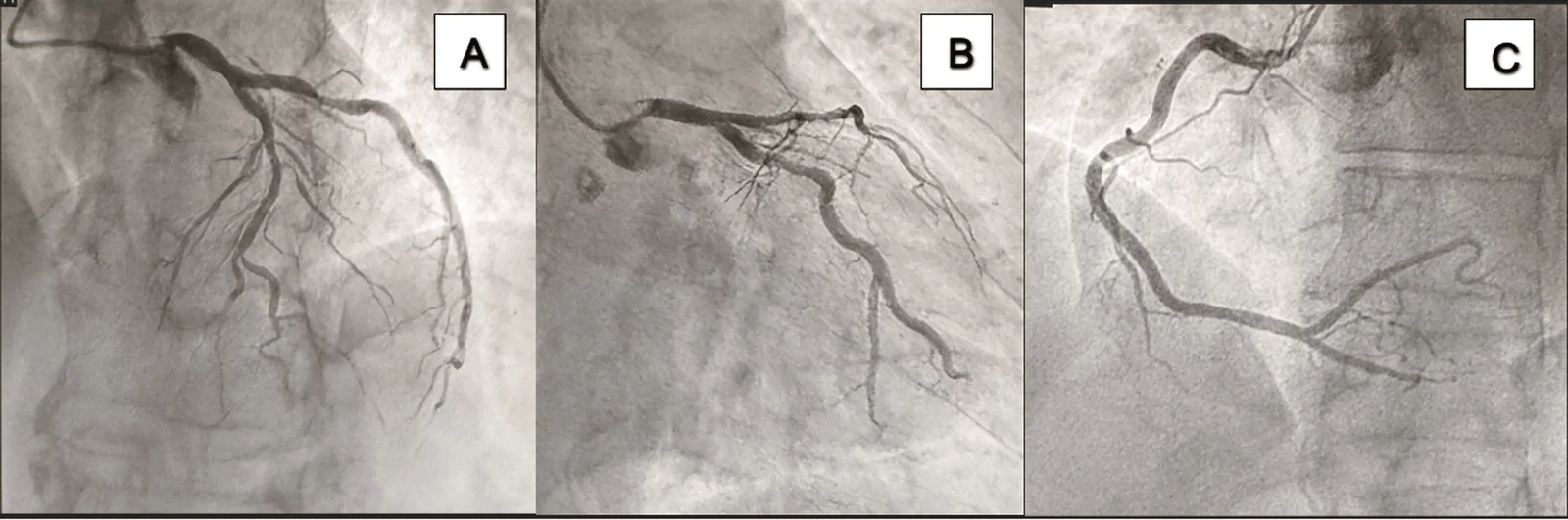
Coronary angiography Panels A and B show the left coronary system in complementary projections, with only minor non-obstructive irregularities. Panel C shows the dominant right coronary artery without significant stenosis. No culprit lesion or angiographic thrombus was identified.

Given the immediate relationship between the hornet sting, anaphylactic shock, and the onset of chest pain, together with transient ischemic ECG changes, troponin elevation, and the absence of a culprit coronary lesion, the diagnosis of type I Kounis syndrome was made. The minor coronary irregularities were considered incidental and not responsible for the acute event.

The subsequent hospital course was uncomplicated. There was no recurrent chest pain, dyspnea, heart failure, clinically significant arrhythmia, or conduction disturbance. Troponin progressively decreased from 208 to 67 ng/L. Episodes of nocturnal bradycardia were considered most likely related to the patient's known obstructive sleep apnea. Follow-up cardiac magnetic resonance imaging showed no significant abnormality and no pathological late gadolinium enhancement. The patient was discharged on aspirin 75 mg once daily and atorvastatin 40 mg once daily because of the mild non-obstructive coronary atherosclerosis seen on angiography.

## Discussion

Kounis syndrome is best understood as a coronary manifestation of systemic hypersensitivity rather than a coincidental association between allergy and acute coronary syndrome. Mast-cell activation is central to the process. Histamine, leukotrienes, tryptase, chymase, thromboxane, and platelet-activating factor can alter coronary vascular tone, activate platelets, and, when atherosclerosis is present, contribute to plaque instability [2,3].

Our patient was diagnosed with type I Kounis syndrome. Although angiography showed minor irregularities of less than 30%, there was no significant obstructive disease or culprit lesion. The event was characterized instead by transient ischemia: chest pain began during the allergic reaction, ST-segment depression was documented during the acute episode and subsequently regressed, left ventricular systolic function remained preserved, and follow-up cardiac magnetic resonance imaging was normal. Recent descriptions of type I Kounis syndrome include patients with normal or near-normal coronary arteries, making minimal non-obstructive irregularities compatible with a predominantly vasospastic phenotype [2,3,5].

The increase in troponin does not argue against type I disease. Coronary spasm can be sufficiently intense or prolonged to cause myocardial injury even when the artery appears normal after the episode has resolved. Vu et al. described a type I Kounis syndrome with ischemic electrocardiographic changes and elevated cardiac biomarkers despite the absence of significant coronary stenosis [6]. This pattern is similar to our patient, in whom troponin peaked at 208 ng/L while echocardiography showed no regional wall-motion abnormality.

The hornet sting provides a plausible and well-recognized trigger. Hymenoptera-associated Kounis syndrome has been reported after both bee and hornet stings, with cardiovascular manifestations occurring shortly after exposure [4]. In our case, the close temporal relationship was particularly suggestive: the chest pain began during the anaphylactic reaction, and ischemic ECG changes were documented during the same acute episode.

The most important diagnostic question was whether epinephrine contributed to the coronary event. Epinephrine remains the first-line, life-saving treatment for anaphylaxis, but its adrenergic effects can increase myocardial oxygen demand and may promote coronary vasoconstriction, platelet activation, or arrhythmias, particularly at high doses or when given intravenously [5,7,8]. This creates a genuine diagnostic dilemma when myocardial ischemia occurs during treated anaphylaxis.

However, epinephrine should not automatically be blamed simply because it was administered near the time of the cardiac event. Granata et al. emphasized that temporal proximity alone does not establish causality and that Kounis syndrome may already be evolving before treatment is given. They also noted the lack of robust evidence that standard therapeutic intramuscular epinephrine worsens Kounis syndrome, whereas delayed treatment of severe anaphylaxis is a recognized danger [7].

Hamideh et al. identified the onset of cardiac symptoms before epinephrine as a feature favoring anaphylaxis-related coronary ischemia rather than a superimposed epinephrine-induced acute coronary syndrome [5]. A subsequent influence of epinephrine on coronary tone cannot be completely excluded, but it is unlikely to explain the initiation of the chest pain.

The case reported by Ongun et al. illustrates a different sequence. Their patient developed acute coronary manifestations after an inadvertent intravenous epinephrine bolus, making catecholamine-induced vasospasm an important competing explanation [8].

Takotsubo syndrome was another relevant differential diagnosis. Severe anaphylaxis can produce a substantial endogenous catecholamine surge, and exogenous catecholamines may amplify this response. The interaction among adrenaline, Takotsubo cardiomyopathy, anaphylaxis, and Kounis syndrome has been described as the ATAK complex [9]. In our patient, Takotsubo syndrome was considered unlikely because echocardiography during the acute episode showed preserved left ventricular systolic function without regional wall-motion abnormalities. The subsequent normal cardiac magnetic resonance examination provided additional support against persistent structural myocardial injury.

The diagnostic work-up had limitations. Specific markers of mast-cell activation, particularly serum tryptase, were not obtained during the acute episode. Tryptase is most informative when measured early because of its short circulating half-life [9]. In addition, coronary spasm was not directly visualized because angiography was performed after the symptoms and ECG changes had resolved. No intracoronary provocation testing, optical coherence tomography, or intravascular ultrasound was performed. The route of the initial epinephrine dose was not available in the transfer documentation. These limitations mean that the vasospastic mechanism was inferred from the clinical sequence rather than demonstrated directly.

Despite these limitations, the overall pattern was coherent. Management of Kounis syndrome requires attention to both the allergic reaction and the coronary presentation. Epinephrine should not be withheld when it is required for life-threatening anaphylaxis, although cardiovascular monitoring is appropriate. Antihistamines and corticosteroids are commonly used for the hypersensitivity component, while nitrates or calcium-channel blockers may be considered when vasospasm persists and blood pressure permits [7,9]. Coronary angiography is particularly useful when biomarkers are elevated or ischemic ECG abnormalities persist because it helps distinguish vasospasm from a plaque-related acute coronary syndrome or coronary thrombosis.

## Conclusions

Type I Kounis syndrome should be considered when objective signs of myocardial ischemia occur during an allergic or anaphylactic reaction, including after a Hymenoptera sting. Transient ST-segment abnormalities and troponin elevation can occur despite preserved ventricular function and the absence of significant obstructive coronary disease.

Epinephrine remains essential in life-threatening anaphylaxis, but careful cardiac evaluation is warranted when ischemic symptoms accompany the allergic event.
